# Prediction or causality? A scoping review of their conflation within current observational research

**DOI:** 10.1007/s10654-021-00794-w

**Published:** 2021-08-15

**Authors:** Chava L. Ramspek, Ewout W. Steyerberg, Richard D. Riley, Frits R. Rosendaal, Olaf M. Dekkers, Friedo W. Dekker, Merel van Diepen

**Affiliations:** 1 Department of Clinical Epidemiology, Leiden University Medical Center, Leiden, The Netherlands; 2 Department of Biomedical Data Sciences, Leiden University Medical Center, Leiden, The Netherlands; 3 Centre for Prognosis Research, School of Medicine, Keele University, Keele, UK

**Keywords:** Causality, Etiology, Prediction, Methodology, Epidemiology, Scoping review

## Abstract

Etiological research aims to uncover causal effects, whilst prediction research aims to forecast an outcome with the best accuracy. Causal and prediction research usually require different methods, and yet their findings may get conflated when reported and interpreted. The aim of the current study is to quantify the frequency of conflation between etiological and prediction research, to discuss common underlying mistakes and provide recommendations on how to avoid these. Observational cohort studies published in January 2018 in the top-ranked journals of six distinct medical fields (Cardiology, Clinical Epidemiology, Clinical Neurology, General and Internal Medicine, Nephrology and Surgery) were included for the current scoping review. Data on conflation was extracted through signaling questions. In total, 180 studies were included. Overall, 26% (n = 46) contained conflation between etiology and prediction. The frequency of conflation varied across medical field and journal impact factor. From the causal studies 22% was conflated, mainly due to the selection of covariates based on their ability to predict without taking the causal structure into account. Within prediction studies 38% was conflated, the most frequent reason was a causal interpretation of covariates included in a prediction model. Conflation of etiology and prediction is a common methodological error in observational medical research and more frequent in prediction studies. As this may lead to biased estimations and erroneous conclusions, researchers must be careful when designing, interpreting and disseminating their research to ensure this conflation is avoided.

**Supplementary Information:**

The online version contains supplementary material available at 10.1007/s10654-021-00794-w.

## Introduction

From an epidemiological perspective, clinical studies are often classified as having either a descriptive, etiological or predictive aim. In the current study we focus on etiology and prediction research. In etiological research the typical aim is to uncover the causal effect of a specific exposure (factor) on an outcome. The results generally help us answer ‘what if’ questions about treatment or management and are imperative in furthering our understanding of the mechanisms of disease. The gold standard to do so is traditionally a randomized experiment. However, this is often not feasible and advancements have been made towards undertaking causal inference from observational data [1]. A crucial part of observational causal studies is correction for confounding (variables which influence both the exposure and outcome and muddle the causal relationship). The data itself cannot tell us which variables give confounding, knowledge and assumptions on the underlying causal structure is necessary.

In prediction research, the goal is a model that utilizes multiple factors (‘predictors’) in combination to accurately predict an outcome in individuals to assist in diagnosis or prognosis. This is usually irrespective of whether included predictors are causal or not. The main focus of prediction model studies is the overall predictive or diagnostic performance of the model which should also be assessed in new patients (validation) [2]. A prior step to model development may be to identify novel predictors that have added predictive value when added to known predictors [3]. For a more in depth explanation on etiology and prediction research see the supplemental material. In the case of counterfactual prediction modelling (which answers ‘what if’ questions on prognosis related to interventions) prediction and etiology intentionally collide [4, 5]. Though an important advancement, such studies are rare, require specialist techniques and are not the focus of this article [6].

Both etiological and prediction studies may be performed on the same observational data, but the underlying research question, methods and interpretation of results are usually different [7]. Unfortunately, when reading and reviewing the medical literature, we have found that these aims, methods, results and interpretations are often confused, leaving us with studies that no longer answer a clear etiological or prediction question and may be misinterpreted. In consultations with researchers, we’ve noticed that the distinction between prediction and etiology is often not clearly stipulated and therefore not considered in the research question or proposed statistical approach. The century-old decree that causal inference cannot be made from observational data has resulted in many causal observational studies using vague terminology and an apprehension to tackle causal questions explicitly [8]. The conflation between causal research and prediction research is also common within statistics and data science [9, 10]. Although conflation of etiology and prediction appears anecdotally to be frequent in medical research, the extent of this is unknown. If the frequency and consequences of this conflation are better understood, this may promote change and awareness in medical research practice and scientific education.

Therefore, our research aim is to quantify the frequency of conflation between etiological and prediction goals, analysis approach and interpretation in observational studies from various medical research fields. Furthermore, we aim to discuss common mistakes underlying this conflation, elaborate on the hazards that lie in these mistakes, and provide recommendations on how to recognize and avoid them.

## Methods

### Data sources and study selection

In order to include a wide range of clinical observational studies, journals from the following six medical fields were included: Cardiology, Clinical Epidemiology, Clinical Neurology, General and Internal Medicine, Nephrology and Surgery. The top-ranked journals from these medical fields, according to the 2018 Clarivate Journal Citation Reports (JCR), were screened. To include Clinical Epidemiology journals we used the JCR category ‘Public, environmental & occupational health’ and for Nephrology the JCR category ‘Urology & Nephrology’, for the other medical fields the identically named JCR categories were referenced. Eligible studies were identified by examining the table of content from the January 2018 issue(s), starting with the journal with the highest impact factor per medical field. A total of 30 studies were included per medical field, and journals from each field were added until this number was reached.

The titles, abstracts and full-text studies in the original article section of the various journals were screened by CLR. Original clinical observational studies (etiological, prognostic and diagnostic) conducted on humans were included. We excluded the following types of research: trials, intervention studies, experiments, descriptive studies, fundamental research, genome wide association studies, methodological studies, systematic reviews, meta-analyses, qualitative studies, case-series, impact assessment studies, simulation studies, counterfactual prediction and cost-effectiveness studies.

### Data extraction

General study characteristics were extracted and each study was classified as being conflated or not, using developed signaling questions (see below). Data-extraction of included studies was performed by CLR. When there was uncertainty on how to score a study, MvD assessed this study independently and the study was discussed by CLR and MvD, if necessary a third assessor was consulted (FWD).

### Assessing conflation of etiology and prediction

To identify conflation, a list of unique study characteristics for both etiology and prediction was developed by CLR, MvD and FWD in an iterative fashion. Conflated studies from personal libraries were discussed and classified into themes and domains of conflation. Previous work on this topic was used to help identify key etiological and prediction characteristics and the STROBE (Strengthening the Reporting of Observational studies in Epidemiology) and TRIPOD (Transparent Reporting of a Multivariable Prediction Model for Individual Prognosis or Diagnosis) guidelines were consulted [7, 11–13]. The developed list of characteristics of etiological and prediction research is shown in Box 1. This list is not exhaustive, but contains key characteristics that belong to either etiological or prediction research (but not to both), are unrelated to the research topic and are relatively easy to assess.

**Box 1:** : Etiology versus prediction key characteristics

	Etiology	Prediction
Research question	Objective is to find a causal relation between exposure(s) and outcome(s)	Objective is to predict or diagnose outcome or improve prediction of an outcome in individuals
Statistical approach	Controls for confounding or mediation analysis, using knowledge and assumptions of causal structure and pathways	Develop and/or validate a multivariable model that contains variables (predictors) based on their ability to predict/diagnose the outcome and usability in practice
Presentation of results	Relative risk or risk difference given the exposure, minimizing bias	Measures of the predictive or diagnostic performance of the multivariable model (e.g. discrimination and calibration)
Discussion and interpretation of results	Causal interpretation and/or recognition limitations that preclude causal inference	Proposed use of the prediction model, for example for risk stratification or prediction of prognosis/diagnosis on an individual level, and/or limitations that preclude use (e.g. poor calibration, need for further validation)

Based on the key characteristics, signaling questions were developed to help identify conflation in various domains. The final signaling questions are shown in supplemental table S1. They were designed so that if any question in both the etiology and prediction column is answered by ‘yes’, this flags the potential for conflation. A single study might have both an etiological and prediction aim and therefore contain characteristics from both study types, and, if correctly performed, such studies were classified as containing both etiology and prediction without conflation.

The use of data-driven methods for confounder selection in etiological studies was considered conflation, unless stated that these covariates were pre-selected based on the causal structure (so as to only include potential confounders). If a study reported adjustment for a list of variables (without further clarification on what these variables were or how they were selected), the statistical approach was labelled as unclear; it didn’t contribute to the classification of etiology or prediction. Causal interpretation of predictors from a prognostic model (in which causal structure was not considered) was deemed conflation. Finally, the reporting of hazard ratios or odds ratios in the results section of a prediction model study was not considered conflation, unless stated that these were effect estimates in which bias was minimized by correcting for confounders or by accounting for confounding through the study design.

### Statistical analysis

Descriptive statistics were used to summarize findings. Depending on their distribution, continuous characteristics are presented as mean with standard deviation (SD) or median with interquartile range (IQR). Dichotomous and categorical variables are summarized by reporting proportions. When appropriate a standard error (SE) or 95% confidence interval (CI) was added. The association between journal characteristics and conflation was quantified in univariate binomial regression analysis and presented as an odds ratio (OR) with CI. Statistical analyses and graphs were computed in R version 3.6.1.

## Results

### Included studies

A total of 421 studies were screened for inclusion based on their title, abstract or full-text. Finally, 180 observational studies in humans were included for the current review; 30 studies were included from each of the 6 considered medical fields (Cardiology, Clinical Epidemiology, Clinical Neurology, General & Internal Medicine, Nephrology and Surgery). See supplemental figure S1 and table S2 for a flowchart of the study selection and list of journals from which studies were included. Each included study was classified as etiology, prediction or both. Subsequently using the signaling questions, it was determined whether each study contained conflation between etiology and prediction. The second assessor was consulted on 15 studies. In Table 1, quotes from three included studies that were conflated are shown as an example of study assessment. These quotes exemplify how conflation may arise in observational studies. Table S3 shows the classification for each included study.

**Table 1:** Three examples of observational studies included in this review are shown

	Example 1 [14]	Example 2 [15]	Example 3 [16]
Research question	*“The aim is to determine the impact of vascular disease burden on longer-term transplant and patient survival after kidney transplantation”*	*“The aim of the present study was to assess predictors of a combined atherosclerotic cardiovascular end point”*	*“…*to develop a risk score that predicted the 5-, 10-, and 20-year individual dementia risk in older individuals*”*
Assessment	The objective seems to be causal	The objective is fitting for a predictor finding study	The objective is to develop a risk score to improve prediction of an outcome
Statistical approach	*“Covariates associated with the outcome with P* < *0.10 in unadjusted analyses were included in multivariable-adjusted analyses”*	*“All variables with a P* < *0.1 for univariate association with the atherosclerotic end point were included in a backwards stepwise Cox regression model to obtain the independent risk factors”*	*“We first used univariate models for each candidate risk factor. Following this step, we employed a multivariate model to estimate the coefficients of selected significant risk factors”*
Assessment	Co-variates selected based on ability to predict the outcome, fitting for a prediction study	Co-variates selected based on ability to predict the outcome, fitting for a prediction study	Co-variates selected based on ability to predict the outcome, fitting for a prediction study
Presentation of results	*“Recipients with vascular diseases were at increased risk for death by at least 1.4 times compared with recipients without vascular disease (adjusted HRs of 1.40).”*	*“In multivariable analysis age, male sex, diabetes and prevalent CVD significantly predicted the atherosclerotic end point”*	*“Age, marital status, BMI, stroke, diabetes, ischemic attacks, and cancer were found to be independently predictive of event risk in the final multivariate model and were used to construct the risk algorithm.” “The C-statistic of the final model was 0.716”*
Assessment	Effect measure given, inviting etiological interpretation	Unclear; the current wording could be found in both a prediction or etiological study	Risk score performance measure given, fitting for prediction study
Discussion and interpretation of results	*“Kidney transplant recipients with vascular disease experienced a survival disadvantage” “Although adjustments were done for multiple confounding factors, there were likely to be unmeasured residual confounders”*	*“Markers of inflammation and oxidative stress were significant predictors, future studies should evaluate whether they may be targets for novel treatment strategies” “However, residual confounding due to measurement error, unmeasured risk factors and the lack of adjustment for time-varying covariates constitutes important limitations”*	*“The dementia risk score permits prediction of an individual’s risk of developing dementia” “…many risk factors we have examined represent modifiable health and lifestyle behaviors.” “Higher BMI demonstrated some protective effects in this study.” “This risk estimate system … helps individuals to identify their potential risk profile and prevent or delay the future incidence of dementia”*
Assessment	Etiological interpretation. Authors intended to correct for all measured confounders	Etiological interpretation by identifying modifiable risk factors and considering residual confounding	Both predictive and etiological interpretation. The potential causal mechanism between each predictor and the outcome is discussed
Overall assessment	Conflated: Mainly etiological study with conflation in methods by selecting ‘confounders’ based only on their predictive ability (p-values)	Conflated: Mainly prediction study, yet a causal conclusion is made from a data-driven predictive model and residual confounding is mentioned as limitation	Conflated: Prediction model development study with causal interpretation of predictors

Each of these studies contains conflation and quotes from each domain of the article are given that exemplify this. The table should be read vertically

Some quotes are paraphrased slightly to shorten them and abbreviations are written full-out

### Frequency of conflation and study characteristics

**Fig. 1:**
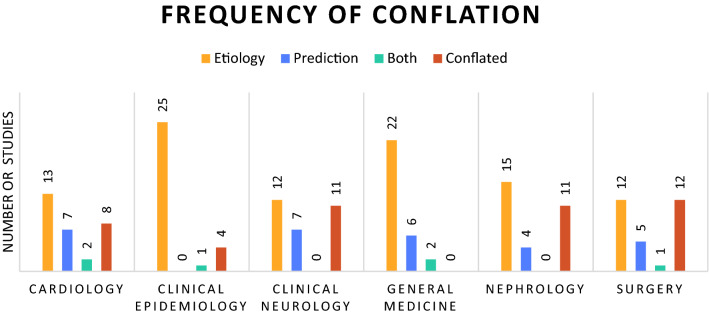
Number of studies included by medical field and assessment; etiological, prediction, both (dual) or conflated

**Fig. 2:**
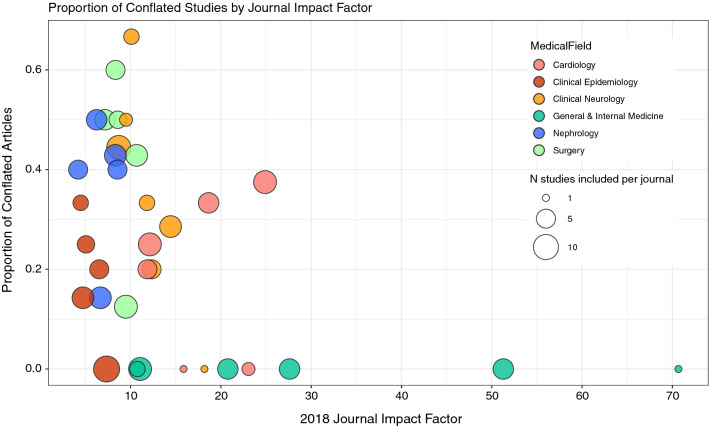
conflation proportion by journal impact factor and medical field. Each bubble represents 1 included journal, the size of the bubble corresponds to the number of articles included from this journal. Spearman’s correlation coefficient between conflation and impact factor is − 0.13 (*p* value 0.08)

Out of 180 studies, 46 were classed as conflated (26%, 95% CI 19–33%). In total, 127 studies (71%) were classified as etiological, 47 (26%) as prediction and 6 (4%) as both prediction and etiology. From the etiological studies 28 (22%) contained conflation and from the prediction studies 18 (38%) contained conflation. In Fig. 1 the classification of studies is shown per medical field, the proportion of conflation ranges from 0 to 40%. In our sample from General & Internal Medicine journals there was no article with conflation between etiology and prediction (30 in total), Clinical Epidemiology journals showed the second-least amount of conflation with 4 conflated articles (13%). In Fig. 2 each included journal is plotted according to its impact factor and proportion of conflated articles. In univariate regression impact factor and conflation were significantly associated; with every point increase in impact factor the odds of conflation decreased (OR 0.95, 95% CI 0.90–1.00).

General characteristics of the included studies are shown in Table 2. An epidemiology department was less frequently listed in the author affiliations of conflated articles (22% vs. 43%; OR 0.36, 95% CI 0.17–0.79). In total only 11 studies reported adherence to a reporting guideline and this reporting was less frequent in conflated articles (2% vs. 7%), odds ratio 0.28 (95% CI 0.03–2.21). The referenced reporting guidelines were STROBE (n = 8), RECORD (n = 2), STARD (n = 1) and PRISMA (n = 1).

**Table 2:** Characteristics of included studies

	Total N = 180	Not conflated N = 134	Conflated = 46
Population			
General population	56 (31%)	51 (38%)	5 (11%)
Primary care	12 (7%)	10 (8%)	2 (4%)
Secondary/tertiary care	112 (62%)	73 (55%)	39 (85%)
Median impact factor (IQR)	9.5 (7.3–12.3)	10.7 (7.3–14.4)	8.6 (7.2–11.8)
Affiliated epidemiology department	68 (38%)	58 (43%)	10 (22%)
Affiliated statistics department	46 (26%)	33 (25%)	13 (28%)
Adherence to reporting guideline	11 (6%)	10 (8%)	1 (2%)

### Types of conflation identified

**Fig. 3:**
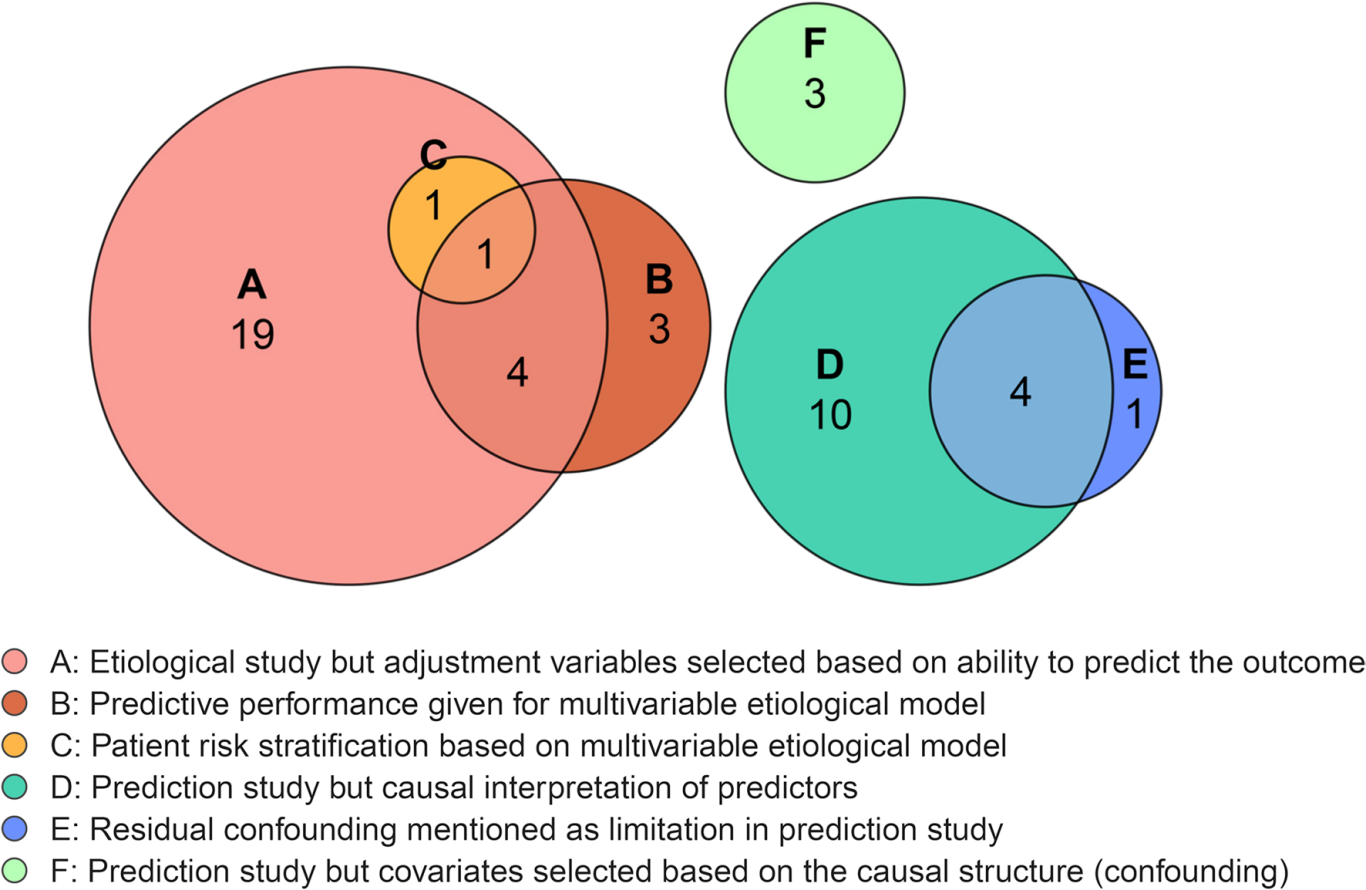
Types of conflation. Type A = 25 studies, type B = 8 studies, type C = 2 studies, type D = 14 studies, type E = 5 studies, type F = 3 studies

In Fig. 3, different types of conflation are shown. We identified six main forms of conflation, of which multiple may be present in the same study. The most frequent form of conflation was the inclusion of covariates based on their ability to predict the outcome without taking the causal structure into account, in an otherwise etiological study (type A). This was observed in 25 out of 127 etiological studies (20%) and always entailed data-driven selection of confounders, based either on univariate associations or stepwise selection procedures. Another frequent mistake (n = 8) in causal research, was the presentation of predictive performance results, such as an AUC or calibration, for the adjusted model (type B). Additionally, two etiological studies proposed risk group stratification based on their multivariable causal model (type C).

In studies that were predictive in aim, the most frequent mistake was a causal interpretation of identified predictors and their estimate of effect (type D). This occurred in 14 studies out of 47 prediction studies (30%). These studies often described which predictors were modifiable and then suggested changing these predictors to improve prognosis. Furthermore, residual confounding was frequently (n = 5, 11%) recognized as limitation in prediction research (type E). Finally, three prediction studies selected predictors by restricting to causal factors or confounders, without having a counterfactual prediction goal.

## Discussion

In this scoping review which sampled observational studies from top journals in 6 medical fields, we found that conflation between etiology and prediction occurred in 46 out of 180 included studies (26%) and was more common in prediction studies. The frequency of conflation varied per medical field and journal impact factor, with less conflation in general & internal medicine and epidemiology journals. In causal research, the most frequent type of conflation was selecting adjustment covariates based on their ability to predict the outcome, rather than based on causal reasoning or pathways. In prediction studies, the most frequent form of conflation was a causal interpretation of predictor effects, instead of referring to their added value for risk prediction or overall model performance.

The identified conflation between etiology and prediction could easily lead to incorrect estimations and erroneous conclusions. If confounders are not accounted for in etiological research, effect estimates are most likely incorrect [17]. Specifically, in medicine it is important to determine causal factors correctly, as they might go on to be the target of novel pharmacological studies, be incentive for randomized controlled trials, or be incentive to change clinical patient care. If incorrect causal claims are made based on prediction models, this could have similarly detrimental effects on patients and further research efforts [9]. In our scoping review, 30% of prediction studies interpreted included predictors causally for instance by suggesting modification of a predictor to improve a patient’s prognosis. This is a dangerous misinterpretation; since predictors in no way need to be causally associated with the outcome, these studies cannot conclude that an individual’s prognosis would change if these predictors were to be modified. Imagine a prediction model of mortality, in which medication use is a predictor associated with a higher risk of death. Though medication use is modifiable, this does not mean that individuals should discontinue their medication in order to live a longer life. Though this example is rather obvious, similar mistakes frequently occur. For instance, an included study presenting a dementia risk score, concludes that a high BMI is protective. These conclusions may mislead readers into thinking obesity has health benefits [16]. Other identified conflation types, such as the presentation of performance measures for causal models or the recognition of residual confounding as limitation in prediction studies, probably have fewer directly harmful effects but constitute poor research methodology. Performance measures have a very limited role in etiological research and the concept of confounders or residual confounding is not appropriate in prediction studies that are not designed or aimed at uncovering a causal effect. We believe reducing conflation between etiology and prediction will improve the quality of observational research and lead to better and more efficient science.

Many excellent papers and books have been published on how to conduct both etiological and prediction research on observational data [18–23]. If these guidelines are followed precisely, conflation between etiology and prediction is unlikely. However, few studies explicitly tackle the differences between etiology and prediction. A study from Zalpuri et al. from 2012 surveyed 435 attendees of an international transfusion conference on the interpretation of a multivariable stepwise logistic regression model. In total, 40% of attendees thought that a stepwise model was a valid method to adjust for confounding and 60% of attendees agreed with a causal interpretation of a stepwise prediction model [24]. An important paper by Shmueli does discuss explaining versus prediction from a statistical viewpoint and concluded that the statistical literature lacks a thorough discussion of differences between prediction and causality [10]. A recent paper by Hernan et al. entitled ‘a second chance to get causal inference right’ contains a plea to data scientists to integrate causal and prediction research questions (and their differences) in their curricula and analysis framework [9]. In previous work we also addressed differences between prediction and etiology and discussed related common pitfalls that arise [7]. The current study has empirically confirmed these pitfalls, quantified how frequent they are, and identified additional types of conflation.

There are various factors that may contribute to conflation between etiology and prediction. We have formulated some general recommendations for researchers, research-institutes, journals and policy-makers (Table 3) to ensure a clear distinction. For researchers, one of the most important recommendations is to clearly define the research question (including whether it is causal, predictive or descriptive). If the research question is unclear this ambiguity frequently continues throughout the rest of the article [25]. Once the aim is clear we suggest consulting appropriate reporting and methodological guidelines as well as methodological experts. For observational causal studies we recommend the STROBE guideline and the accompanying explanation & elaboration paper [18]. For diagnostic and prognostic prediction studies TRIPOD is recommended [19, 20]. Terms such as ‘predictor’ are frequently used in both causal and predictive studies. Though this in itself does not constitute conflation, it may cause uncertainty on the aim of the study. We would suggest reserving the terms ‘risk factor’, ‘causal factor’, ‘exposure’ and ‘confounder’ for causal research and the terms ‘predictor’, ‘prognostic factor’, and ‘prediction’ for prediction studies. The word causation is often avoided in observational studies, and sometimes even forbidden by journals [8]. Though no study can definitively prove causation, lack of clarity regarding the research goals has negative effects on the quality of observational research [8]. Finally, it would be beneficial for journals to include methodological experts in the peer review and editorial process. Our signaling questions can be used by reviewers or in systematic reviews to quantify the risk of conflation between etiology and prediction in observational studies.

**Table 3:** Recommendations on how to avoid conflation

Recommendations for researchers	
1	Clearly define the research question and consider whether the aim is causal, predictive, diagnostic or descriptive
2	Be mindful of frequent mistakes that cause conflation between etiology & prediction and distinguish between the two by using appropriate terminology
3	Consult methodological experts as well as reporting and methodological guidelines (e.g. STROBE, TRIPOD, STARD, REMARK)
Recommendations for universities, journals & policy-makers	
1	Work on improving education on prediction research and the distinction between prediction & etiology, including the promotion of distinct terminology for prediction and etiological research
2	Promote the use of reporting and methodological guidelines
3	Include methodological expert as peer-reviewers and/or editors

Machine learning techniques are methodological advancements that have large potential in prediction research to analyze complex data structures [26]. However, the lack of transparency and black box nature of these methods complicates independent validation, updating and implementation. Therefore, it is imperative that such studies adhere to the same methodological guidelines as regression based prediction studies [26]. The more recent proposed use of machine learning in causal inference is not straightforward and can easily introduce conflation. Similar to data-driven selection methods, machine learning algorithms cannot distinguish mediators from confounders or recognize bias; the researchers’ knowledge and input on the causal structure remains crucial [27].

Though we have argued that variables in a prediction model should not be interpreted causally, many prediction models will benefit from including (previously identified) causal factors as predictors. This may help improve transportability of the model to new settings or different populations and can improve credibility and uptake of the model. Some might even go so far as to say that the ultimate prediction model—though unattainable—would contain all and only causal pathways of a condition [28]. On a less philosophical note, there are research questions for which prediction and etiological methods should be combined. The rather new approach termed counterfactual prediction per definition intertwines prediction and causation. Such studies aim to predict an individual’s prognosis for multiple scenario’s, while only one of these scenario’s is observed in the data (per individual). In particular, development datasets will typically observe outcomes in the context of current care, which includes current treatment and monitoring procedures. Thus, counterfactual prediction is then needed to make risk predictions in the hypothetical scenario where individuals receive a different treatment or care. This allows the comparison of multiple predicted risks for various treatment pathways within one individual.[4–6, 29] Such research questions require causal inference methods such as inversed probability weighting when constructing a prediction model. It is worth mentioning that such counterfactual prediction studies are currently rare, and indeed were not encountered in our review, but they should not be seen as undesirable conflation.

The current study has a number of limitations. First of all, we included a limited sample of observational studies from 2018 which might not be representative of other years, journals or medical fields. It is worth noting that all recommended reporting guidelines were published before 2018. The median impact factor of our sample was relatively high (9.5) and it is likely that the amount of conflation is higher in journals with lower impact factors, which was a trend we also observed within our sample. Secondly, the data-extraction was performed by a single researcher and a second assessor was only consulted when the first assessor was unsure. The assessment will invariably contain some subjectivity, particularly whether heavily conflated articles are mainly etiological or predictive in aim. Furthermore, we could not assess whether relevant confounders were included or excluded for all included etiology studies, as this requires subject specific expertise. Importantly, our list of signaling questions is not exhaustive and may not pick up on all conflation. Conflation may not always be reflected in used terminology and studies labelled unclear may actually have been conflated. Additionally, conflation between prediction and etiology is only a small part of assessing a study’s methodological quality. A study which does not confuse prediction and etiology may still be sloppy or incorrect and some elements that we termed conflation, others might call poor etiology or prediction practices. Similarly, not all conflation leads to wrong conclusions. Finally, not all observational clinical research can be classified as either etiology or prediction and we appreciate the broad spectrum of forms that may lie between these two or completely outside of these realms.

In conclusion, undesirable conflation between prediction and etiology is common in medical observational studies and may present an obstacle to scientific progress. Researchers and readers should be mindful of the differences between etiology and prediction, to prevent biased estimates, erroneous conclusions and steering research in an inefficient or wrong direction.

## Supplementary Information

Below is the link to the electronic supplementary material.

Supplementary file1 (PDF 381 kb)

## Data Availability

Data will be made available upon reasonable request to the corresponding author (C. L. Ramspek).
