# Supplementary material for: Prediction or causality? A scoping review of their conflation within current observational research
Source: Eur J Epidemiol. 2021 Aug 15;36(9):889–98. doi: 10.1007/s10654-021-00794-w (PMC8502741; doi:10.1007/s10654-021-00794-w)
Supplement: Supplementary file 1 — Supplementary file1 (PDF 381 kb) [file 10654_2021_794_MOESM1_ESM.pdf]

Supplemental Material for:

Prediction or causality? A scoping review of their conflation within current observational research

Chava L. Ramspek[1], Ewout W. Steyerberg[2], Richard D. Riley[3], Frits R. Rosendaal[1], Olaf M. Dekkers[1], Friedo W. Dekker[1], Merel van Diepen[1]

[1]Department of Clinical Epidemiology, Leiden University Medical Center, the Netherlands.
[2]Department of Biomedical Data Sciences, Leiden University Medical Center, the Netherlands.
[3] Centre for Prognosis Research, School of Medicine, Keele University, Keele, UK.

Background

Etiology explained

Research aiming to explain can be called causal or etiological and has been the cornerstone of modern research. Particularly in the medical field, most studies are causal in nature as they aim to understand why disease occurs and how best to treat it. The golden standard of etiological research has long been considered to be an experiment (e.g. a randomized controlled trial).[1] However, many causal questions cannot be answered with a trial due to, among other things, practical, monetary, and ethical constraints. In recent years causal inference knowledge has expanded greatly and many methods have been developed to approximate causal effects in observational data.[2]

In a well-defined etiological study the researcher is interested in the causal effect of an exposure variable on an outcome. To find the most precise estimate of the causal effect, the exposed and unexposed patients should be (made) as similar as possible for characteristics that may influence the outcome. If not, the presence of confounding will give biased (untrue) causal effects. Confounding is the presence of other characteristics which influence both the exposure and outcome and thereby muddle the causal relationship.[3] A simple example is that grey hair is associated with mortality, however this relationship is completely explained by age, which is a (indirect) cause of both grey hair and death. To describe the causal structure of the study question and adjust for confounding variables, expert knowledge on the subject is essential.[4] The data itself cannot tell us which variables are confounders and which are in the causal pathway (mediators) between exposure and outcome, as the data can only reveal associations and not the direction of effect.[4, 5] When interested in the total causal effect we only want to adjust for confounders, as adjusting for mediators removes part of the exposure effect on the outcome. For instance, when researching the total causal effect of blood pressure on mortality, it is important to adjust for confounders such as age and BMI (as these factors potentially affect both blood pressure and mortality). If we were to select correction factors based on the data, for instance by including all factors univariately associated with the outcome/exposure or by using backward selection procedures, we might also adjust for cardiovascular disease and heart failure (as these factors are associated with both our exposure and outcome in the data). As this factor lies along the causal pathway (hypertension can cause cardiovascular disease and heart failure which in turn may lead to death) we are over-adjusting and we could erroneously conclude that hypertension does not affect mortality risk. However, if we want to look into why hypertension increases the risk of mortality, we may intentionally adjust for mediators, to assess how much of the effect of hypertension on mortality is due to heart failure. In such studies the aim is not to quantify the total causal effect but to disentangle the causal mechanism.

With expert knowledge on the underlying causal structure we can account for confounding in the study design (e.g. through restriction, matching, Mendelian randomization or instrumental variable

analysis) or in the data analysis.[6-8] The most used methods in causal inference are multivariable regression techniques, in which we can calculate the exposure effect conditional on the presence of confounding factors, though more complex analytic methods such as propensity score matching or inverse probability weighting are also employed.[2] Though generally not advisable, data-driven confounder selection may be employed in small datasets, under the condition that the data has been pre-processed to entail that covariates fed into the statistical selection method are only potential confounders and free of mediators.[5, 9, 10]

The hypothesized causal structure should be carefully considered prior to analysis, preferably using causal diagrams (directed acyclic graphs) which were first described in a hallmark paper by Greenland, Pearl and Robbins.[11] Causal diagrams can assist researchers in identifying confounding factors, colliders and covariates that act like instrumental variables. Whilst failing to correct for a confounding factor can introduce bias, conditioning *on* a collider (common effect of exposure and outcome) or instrument can also introduce and amplify bias.[12, 13] The presented results of etiological studies are the risk of the outcome in exposed compared to unexposed patients, with a focus on minimizing bias to obtain the most accurate estimate of the effect of each included factor. This is most often reported as a risk ratio, odds ratio or hazard ratio but may also be shown in terms of absolute risk. These results help us answer 'what if' questions about treatment or management and are imperative in furthering our understanding of health and disease. What if we dyed all grey hair brown, would survival improve? Our age-adjusted result gives us the (rather intuitive) answer: no.

Prediction clarified

In medical research prediction models are either prognostic or diagnostic, where prognostic models generally provide individual risk estimates of a future binary outcome and diagnostic models predict the current presence or absence of a condition.[14] The past decade has seen an almost exponential surge in the number of medical prediction studies.[15] Ideally, prognostic questions are answered in observational prospective cohort studies. Though data from existing trials may be used this is often less favorable as strict selection criteria limit generalizability.[14] Prediction studies are particularly valued for their applied utility, as they may be employed to improve individualized decision-making, selection of high or low-risk patient groups, and personalized medicine.[16]

In essence a prediction model is an algorithm which converts an individual's characteristics into a prognosis or diagnosis. A precursor to prediction modelling may be identifying predictors. Predictor finding studies (or prognostic factor finding studies) don't aim to develop a full prediction model but instead have the goal to identify predictors that contribute added value (over and above existing predictors) to an improved risk stratification or future prediction model.[17] Ideally, the predictive value of these factors is assessed in addition to readily available known predictors that are currently used. What predictor finding studies and prediction model studies have in common, is that they do not aim to uncover causal effects. A good prediction model predicts outcome with the highest accuracy using readily available predictors. These predictors may be any variable associated with the outcome, causally or otherwise. Though expert knowledge (including causal understanding) and previous research help in selecting relevant predictors, accurate prediction models can be built using only the underlying data as input. Such data-driven methods range from as choosing predictors a priori and developing a statistical model that includes all of them; to selection procedures such as backwards selection or those that incorporate penalization such as lasso and elastic net; and to machine learning algorithms such as decision trees, random forests, support vector machines, neural networks).[15]

Importantly, this means that we cannot interpret predictors as causal.[14] Though this sounds rather intuitive, in practice it is tempting to do so. For instance when filling in a prediction model of mortality risk, blood pressure may be a predictor. When calculating the mortality risk for an individual with hypertension, it is compelling to check what would happen to this person's prognosis if we could lower the blood pressure a bit further. However, the difference in prognosis when reporting different blood pressure values does not reflect an actual risk reduction if we modify this predictor. Even if a model included all confounders for the effect of interest and no colliders, we are fundamentally unable to identify individual causal effects and can only identify average causal effects. However, as the prediction model likely contains various mediators (such as heart failure) between blood pressure and mortality and the model may lack important confounders for this association, we absolutely cannot deduce a causal effect. This becomes apparent if we think of a prediction model that includes the use of antihypertensive drugs as predictor. As the use of certain drugs (or lack thereof) can tell us quite a bit about a patient's general health status, it makes sense that such variables may be potent predictors and that, in observational data, the use of antihypertensive drugs are positively associated with mortality. This does not mean that an individual's prognosis would improve if they discontinue their antihypertensive medication, contrarily in all likelihood their prognosis would worsen.

Indeed, the main focus of prediction model studies is not the predictors included in the model or their effect sizes, but rather the overall predictive performance of the model in terms of calibration, discrimination and clinical utility of the model's predicted values in individuals.[18] To this end, measures such as C-statistic (area under the curve), calibration-in-the-large, calibration slope, and net benefit are often used.[19]. Prediction models can help us group patients into high and low risk individuals and personalize medical practice by providing individual diagnosis or prognosis estimates.[20]

Figure S1: flowchart of study inclusion

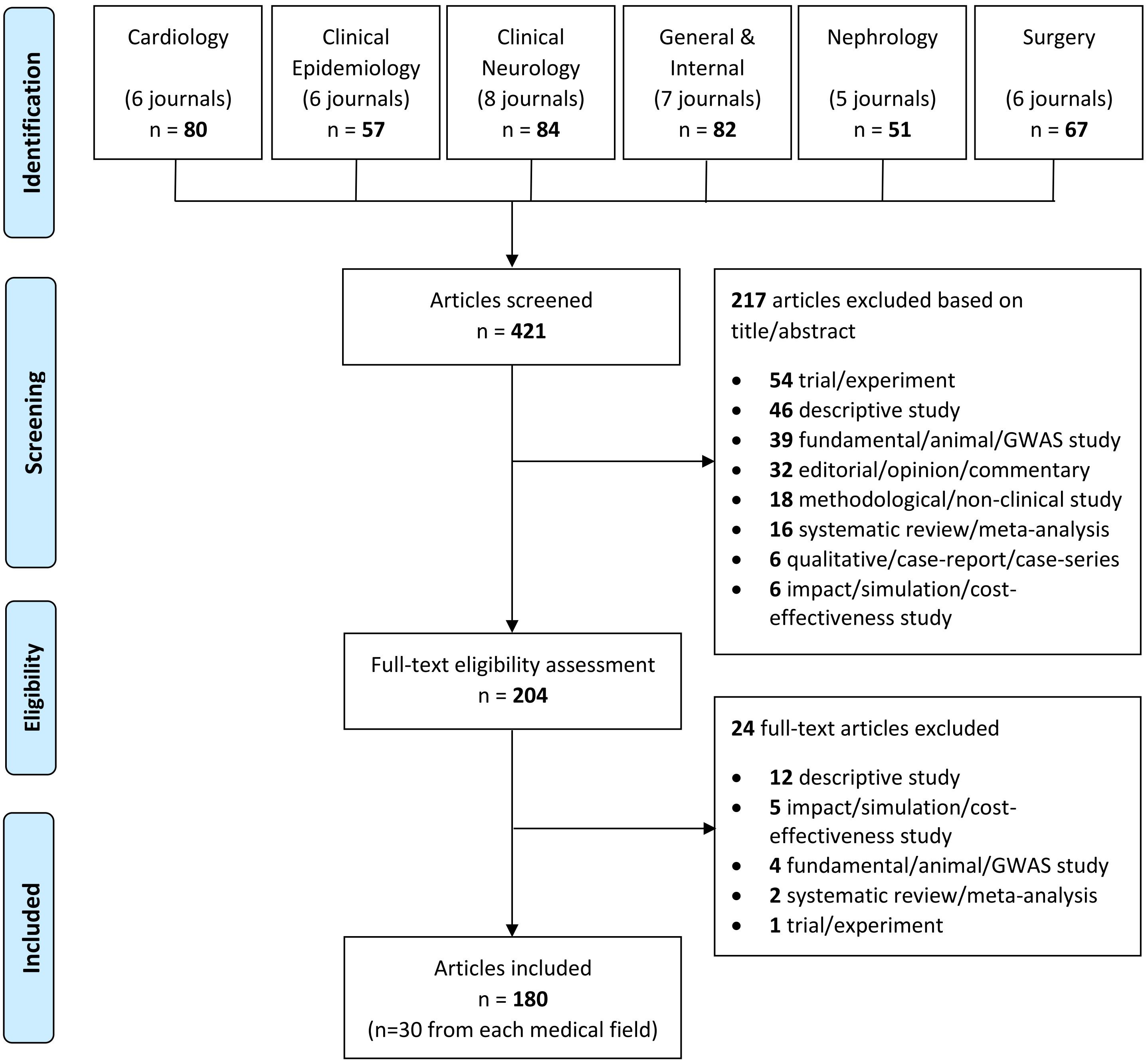

Table S1: Signaling questions for identifying conflation between etiology and prediction in observational studies.

	Etiology	Prediction
**1. Research question**	1.1 Was the objective to find a causal association?	1.2 Was the objective to develop or validate a prediction model?
		1.3. Was the objective to identify predictors or prognostic factors?
**2. Statistical approach**	2.1 Were adjustment covariates included or excluded in multivariable regression based on their role in the causal structures (e.g. as confounder or mediator)?	2.3. Were covariates included in the model based on their ability to predict the outcome (e.g. based on univariate association, backward or forward selection, machine-learning methods)
	2.2. Were methods such as matching, IPW or propensity scores employed, to adjust for differences between exposure groups?	2.4. Were covariates included as predictor based on previous studies or existing prognostic/diagnostic models?
		2.5. Were covariates included as predictor based on clinical expertise?
**3. Presentation of results**	3.1. Were the main results relative or absolute risks in which bias was minimized (for instance by adjusting for confounders or matching)?	3.2 Were performance measures (e.g. AUC, sensitivity, calibration) presented for the multivariable model?
		3.3 Were patients diagnosed or stratified according to risk, based on the multivariable model?
**4. Discussion and interpretation of results**	4.1 Were any of the variables from the multivariable model interpreted in a causal manner?	4.4 Was the multivariable model proposed for risk stratification?
	4.2 Was residual confounding mentioned as limitation of the study or was full confounder adjustment mentioned as strength?	4.5 Was the multivariable model proposed for use in individuals for diagnostic or prognostic purposes?
	4.3 Were interventions that modify risk factors recommended based on the study results?	

Scoring: Each question should be answered by Yes/No/Unclear when assessing a study. If any questions are answered by ‘Yes’ in both the etiological and prediction column, there may be conflation between etiology and prediction.

Table S2: Number of studies screened and included per journal.

Medical field	Journal	N studies screened	N studies included	N studies with conflation
Cardiology	European Heart Journal	14	8	3
Cardiology	Circulation	8	2	0
Cardiology	Journal of the American College of Cardiology	40	6	2
Cardiology	Circulation Research	1	1	0
Cardiology	European Journal of Heart Failure	11	8	2
Cardiology	JAMA Cardiology	6	5	1
Clinical Epidemiology	International Journal of Epidemiology	18	11	0
Clinical Epidemiology	European Journal of Epidemiology	10	5	1
Clinical Epidemiology	Cancer Epidemiology, Biomarkers & Prevention	8	4	1
Clinical Epidemiology	Epidemiology	10	7	1
Clinical Epidemiology	Journal of Clinical Epidemiology	6	0	-
Clinical Epidemiology	American Journal of Epidemiology	5	3	1
Clinical Neurology	The Lancet Neurology	4	0	-
Clinical Neurology	ACTA Neuropathologica	6	1	0
Clinical Neurology	Alzheimer’s & Dementia	9	7	2
Clinical Neurology	JAMA Neurology	11	5	1
Clinical Neurology	BRAIN	17	3	1
Clinical Neurology	Neuro-Oncology	4	3	2
Clinical Neurology	Annals of Neurology	15	2	1
Clinical Neurology	Neurology	18	9	4
General & Internal Medicine	New England Journal of Medicine	15	1	0
General & Internal Medicine	Journal of the American Medical Association (JAMA)	14	6	0
General & Internal Medicine	British Medical Journal	11	6	0
General & Internal Medicine	JAMA Internal Medicine	10	6	0
General & Internal Medicine	Annals of Internal Medicine	6	0	-
General & Internal Medicine	PLOS Medicine	14	8	0
General & Internal Medicine	Journal of cachexia, sarcopenia and muscle	12	3	0
Nephrology	Journal of the American Society of Nephrology	12	5	2
Nephrology	Kidney International	9	7	3
Nephrology	American Journal of Kidney Diseases	11	7	1
Nephrology	Clinical Journal of the American Society of Nephrology	12	6	3
Nephrology	Nephrology Dialysis Transplantation	7	5	2
Surgery	JAMA Surgery	10	7	3
Surgery	Annals of Surgery	24	8	1
Surgery	Journal of Heart and Lung Transplantation	11	4	2
Surgery	Journal of Neurology, Neurosurgery and Psychiatry	9	5	3
Surgery	American Journal of Transplantation	10	6	3
Surgery	Endoscopy	4	0	-

Table S3: Visual representation of scoring per included article. Each row represents one of the included articles. Each article section was scored as fitting for etiology, prediction, unclear, both or conflation. To do so, the signaling questions presented in supplemental table S1 were used. The overall judgement is labeled as conflated if there are contradictory sections (e.g. the methods are fitting for prediction and the discussion for etiology) or if there is conflation within a section.

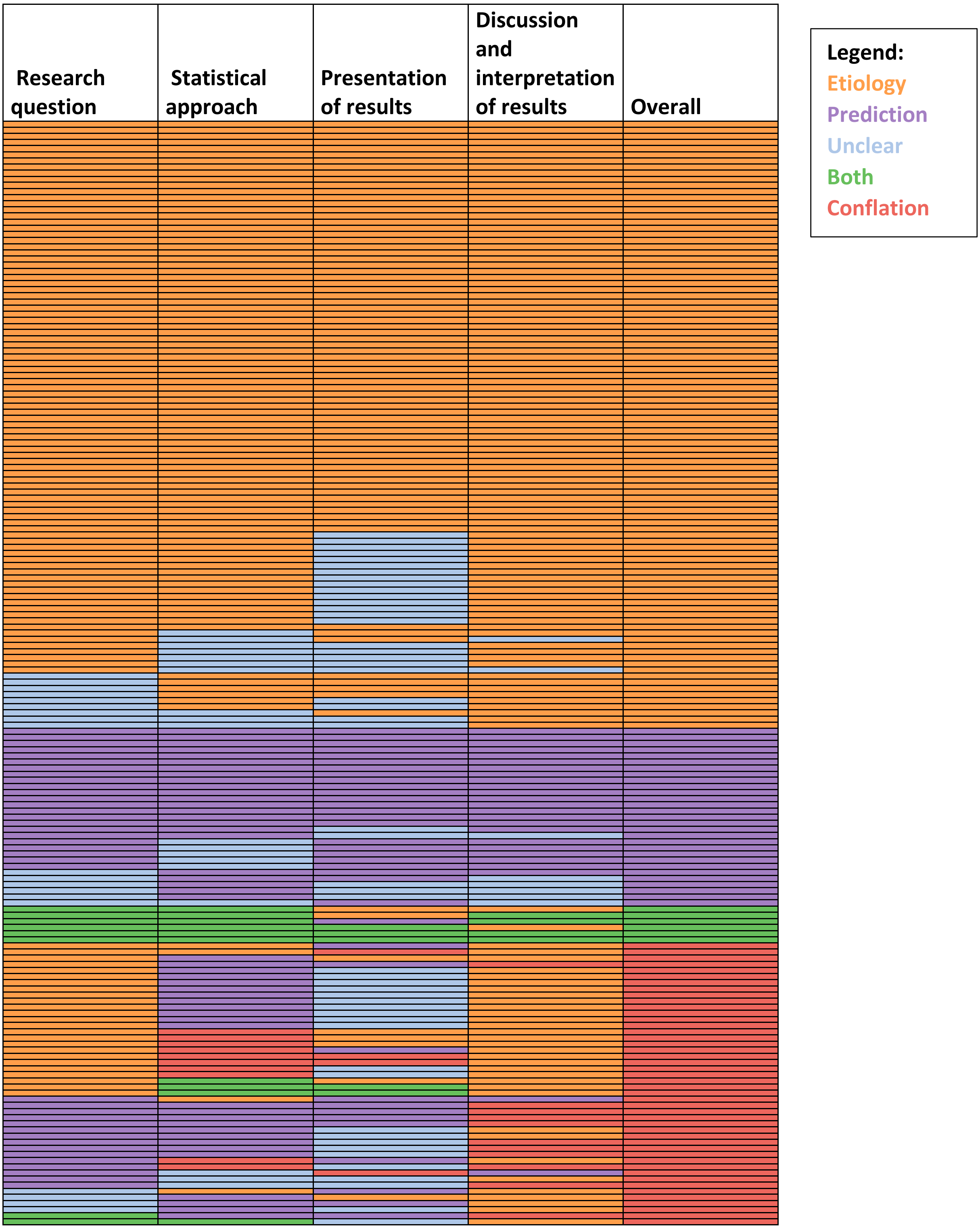

References

1. Jager, K.J., et al., *The valuable contribution of observational studies to nephrology.* Kidney Int, 2007. **72**(6): p. 671-5.
2. Hernán, M.A. and J.M. Robins, *Estimating causal effects from epidemiological data.* 2006. **60**(7): p. 578-586.
3. Jager, K.J., et al., *Confounding: what it is and how to deal with it.* Kidney international, 2008. **73**(3): p. 256-260.
4. Hernán, M.A., et al., *Causal Knowledge as a Prerequisite for Confounding Evaluation: An Application to Birth Defects Epidemiology.* American Journal of Epidemiology, 2002. **155**(2): p. 176-184.
5. VanderWeele, T.J., *Principles of confounder selection.* European Journal of Epidemiology, 2019. **34**(3): p. 211-219.
6. Schmoor, C., et al., *Correction of confounding bias in non-randomized studies by appropriate weighting.* Biometrical journal. Biometrische Zeitschrift, 2011. **53**(2): p. 369-387.
7. Braga, L.H.P., F. Farrokhyar, and M. Bhandari, *Confounding: what is it and how do we deal with it?* Canadian journal of surgery. Journal canadien de chirurgie, 2012. **55**(2): p. 132-138.
8. van Stralen, K.J., et al., *Confounding.* Nephron. Clinical practice, 2010. **116**(2): p. c143-c147.
9. VanderWeele, T.J., *Principles of confounder selection.* Eur J Epidemiol, 2019. **34**(3): p. 211-219.
10. Talbot, D. and V.K. Massamba, *A descriptive review of variable selection methods in four epidemiologic journals: there is still room for improvement.* Eur J Epidemiol, 2019. **34**(8): p. 725-730.
11. Greenland, S., J. Pearl, and J.M. Robins, *Causal diagrams for epidemiologic research.* Epidemiology, 1999. **10**(1): p. 37-48.
12. Pearl, J., *Invited commentary: understanding bias amplification.* Am J Epidemiol, 2011. **174**(11): p. 1223-7; discussion pg 1228-9.
13. Cole, S.R., et al., *Illustrating bias due to conditioning on a collider.* International Journal of Epidemiology, 2009. **39**(2): p. 417-420.
14. Moons, K.G.M., et al., *Prognosis and prognostic research: what, why, and how?* BMJ 2009. Feb 23:**338**:b375.
15. Collins, G.S. and K.G.M. Moons, *Reporting of artificial intelligence prediction models.* The Lancet, 2019. **393**(10181): p. 1577-1579.
16. Shmueli, G., *To Explain or to Predict?* Statist. Sci., 2010. **25**(3): p. 289-310.
17. Riley, R.D., et al., *Prognosis Research Strategy (PROGRESS) 2: prognostic factor research.* PLoS Med, 2013. **10**(2): p. e1001380.
18. Riley, R.D., et al., *Prognosis research in healthcare: concepts, methods, and impact*. 2019: Oxford University Press.
19. Steyerberg, E.W., et al., *Assessing the performance of prediction models: a framework for traditional and novel measures.* Epidemiology (Cambridge, Mass.), 2010. **21**(1): p. 128-138.
20. Hendriksen, J.M.T., et al., *Diagnostic and prognostic prediction models.* Journal of Thrombosis and Haemostasis, 2013. **11**(s1): p. 129-141.
